# Characteristics, Risk Factors, and Adverse Outcomes of Hyperkalemia in Acute-on-Chronic Liver Failure Patients

**DOI:** 10.1155/2019/6025726

**Published:** 2019-02-27

**Authors:** Jun-jun Cai, Kai Wang, Hui-qing Jiang, Tao Han

**Affiliations:** ^1^Department of Gastroenterology, The Second Hospital of Hebei Medical University, Hebei Key Laboratory of Gastroenterology, Hebei Institute of Gastroenterology, Shijiazhuang, China; ^2^Dongzhimen Hospital, Beijing University of Chinese Medicine, Beijing, China; ^3^Sunsimiao Hospital, Beijing University of Chinese Medicine, Tongchuan, China; ^4^Department of Hepatology, Tianjin Third Central Hospital, Tianjin Institute of Hepatobiliary Disease, Tianjin Key Laboratory of Artificial Cell, Tianjin, China

## Abstract

**Background:**

Hyperkalemia is a serious complication in cirrhotic patients. However, the clinical characteristics, risk factors, and its impact on the outcomes in acute-on-chronic liver failure (ACLF) patients remain unclear.

**Methods:**

We retrospectively recruited 650 ACLF patients in this study. The risk factors associated with hyperkalemia and its relationship with 90-day mortality were analyzed using multivariable regression models.

**Results:**

Among 650 patients with ACLF, 12.2% (79/650) had hyperkalemia during hospitalization. Higher admission serum potassium levels and the presence of acute kidney injury (AKI) were independent risk factors for hyperkalemia. The prevalence rates of hyperkalemia in patients with and without AKI were 23.6% and 4.6%, respectively (*P*<0.001). Hyperkalemia was a predictor of mortality in AKI and non-AKI patients. The 90-day mortality rates in non-AKI patients with and without hyperkalemia were 44.4% and 24.7%, respectively (*P*<0.001), and in AKI patients with and without hyperkalemia were 80.3% and 56.6%, respectively (*P*<0.001). Hepatic encephalopathy (HE), gastrointestinal bleeding, AKI, hyperkalemia, elevated total bilirubin (TBIL) and international normalized ratio (INR) values, and higher Model for End-Stage Liver Disease (MELD) and chronic liver failure-sequential organ failure assessment (CLIF-SOFA) scores were independent risk factors for predicting the 90-day mortality in ACLF patients.

**Conclusions:**

Hyperkalemia increases the 90-day mortality in ACLF patients; hyperkalemia is associated with AKI. Patients with both AKI and hyperkalemia had the worst outcome.

## 1. Introduction

Impaired potassium homoeostasis represents one of the commonest electrolyte disturbances. Both hypokalemia and hyperkalemia may have immediate deleterious physiological effects and are consistently associated with adverse outcomes [1]. The risk relationship between potassium levels and adverse outcomes is U-shaped, with the lowest risk at serum potassium of 4-4.5 mmol/L [2, 3]. Patients with advanced cirrhosis frequently have impaired potassium homoeostasis. The prevalence of hyperkalemia in this group of patients is 12%-14%, while that in the general population is 2.1%-7.0% [4–6]. Hyperkalemia may lead to arrhythmia, inhibiting the contraction and ventricular fibrillation and even death [7]. Recently, a prospective, multicenter study demonstrated that all cirrhotic patients with a serum potassium concentration of ≥4.8mmol/L (maximum 5.8mmol/L) died within 1 year [8].

Acute-on-chronic liver failure (ACLF) is a devastating entity characterized by acute deterioration of liver function and one or more extrahepatic organ failures, especially kidney failure [9, 10]. The kidney plays a major role in potassium homeostasis, with kidney impairment being an especially prominent risk factor for hyperkalemia. A recent study showed that the incidence of hyperkalemia is 65% higher among children with acute kidney injury (AKI) compared with those without AKI upon admission [11]. Abnormal intake and excretion/loss of potassium resulting from impairment of renal function may lead to hyperkalemia. Several hormones, including aldosterone, antidiuretic hormone, are involved in potassium metabolism. Other risk factors of hyperkalemia, in combination with renal impairment, include diabetes, cardiovascular disease, and use of pharmacologic agents that affect the potassium regulation, such as diuretic therapy, potassium supplements, and the renin-angiotensin-aldosterone system (RAAS) blockers [12–14]. However, the prevalence, risk factors, and impact of hyperkalemia on the outcome of ACLF patients with or without renal impairment remain poorly defined. Therefore, the present study was to determine the prevalence, risk factors of hyperkalemia, and its impact on the outcome of ACLF patients with or without renal impairment.

## 2. Methods

### 2.1. Materials and Methods

**Figure 1:**
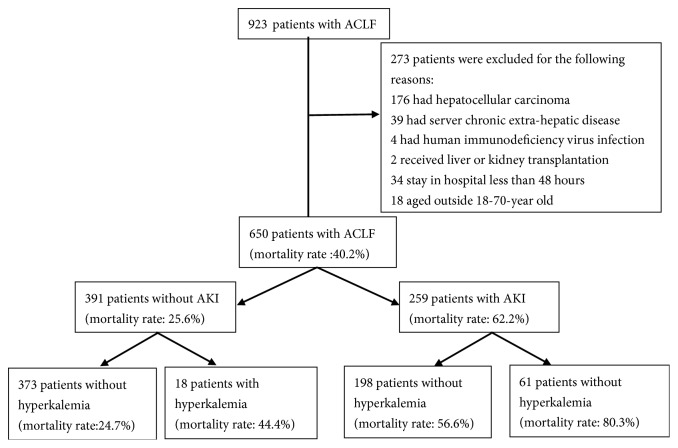
Flow diagram of the study groups selection process. ACLF, acute-on-chronic liver failure; AKI, acute kidney injury.

A flow chart of the patient selection process was presented in Figure 1. We retrospectively recruited 650 patients with ACLF who were hospitalized at Tianjin Third Central Hospital between March 2011 and July 2016. ACLF was defined according to the criteria of Asian Pacific Association for the Study of the Liver (APASL). In brief, acute deterioration of liver function manifested by jaundice (total bilirubin [TBIL]: ≥5mg/dL or ≥85*μ*mol/L) and coagulopathy (international normalized ratio [INR] of prothrombin time: ≥1.5 or prothrombin activity [PTA]: ≤40%]), complicated within 4 weeks with ascites and/or hepatic encephalopathy in a patient with cirrhosis [15]. Exclusion criteria are as follows: (1) patients with hepatocellular carcinoma and nonhepatic neoplasia; (2) immunocompromised patients with human immunodeficiency virus infection; (3) patients with severe chronic extrahepatic disease; (4) patients with a hospital stay of < 48 hours; and (5) patients aged outside 18-70 years old.

Acute kidney injury (AKI) is defined as an increase in serum creatinine value by 0.3mg/dL (26.5*μ*mol/L) within 48 h or percentage by ≥50% from baseline within 1 week according to The Kidney Disease Improving Global Outcomes (KIDGO) criteria [16]. Hyperkalemia was defined as at least one in-hospital serum potassium level measurement >5.5mmol/L. The Model for End-Stage Liver Disease (MELD) score was calculated using the Malinchoc formula [17]. The chronic liver failure-sequential organ failure assessment (CLIF-SOFA) score was calculated according to the criteria of the European Association for the Study of the Liver-Chronic Liver Failure (EASL-CLIF) Consortium [9].

All patients were admitted and received standard supportive treatment. Once recruited, data were collected from the medical records, including patient demographics, vital signs, complete blood count, liver and renal function, INR, electrolytes, and complications of cirrhosis. None of the patients underwent liver transplantation, and none of them were lost to follow-up within the 90-day follow-up period.

### 2.2. Ethics Statement

The study performed in accordance with the principle of the Declaration of Helsinki (2013 revision) and approved by the Institutional Ethical Committee of Tianjin Third Central Hospital. Written informed consents were obtained during the hospital stay. This trial was registered in the Chinese clinical trial registry: ChiCTR1800017991 (http://www.chictr.org.cn/searchchproj.aspx).

### 2.3. Statistical Analysis

Normally distributed variables were expressed as mean ± SD, and nonnormally distributed variables were expressed as a median and interquartile range (IQR). Count and percentages were used to describe categorical variables. Two independent groups were compared using the* t*-test for continuous normally distributed variables and the Mann-Whitney *U* test for nonnormally distributed variables. For categorical variables, comparisons among groups were made using the Chi-squared tests, or Fisher's exact test as appropriate. The Kaplan-Meier method was used to calculate the 90-day survival probability curves, which were compared with the log-rank test [18]. Multivariate logistic regression analyses were fitted to select the main factors associated with the endpoint. Those found to have a* P* value of less than 0.05 on univariate analysis were included in the multiple logistic regression models to evaluate the impact on the presence of hyperkalemia and the 90-day mortality of patients. Two-sided *P* values of < 0.05 were considered statistically significant. All statistical analyses were performed using SPSS software, version 17.0 (SPSS Inc., Chicago, IL, USA).

## 3. Results

### 3.1. Characteristics of Patients

Among 650 ACLF patients, 79 (12.2%) presented hyperkalemia during hospitalization. The characteristics of patients with and without hyperkalemia are summarized in Table 1. Patients with hyperkalemia have a higher baseline serum potassium levels 4.7 (3.7-5.5) mmol/L than those without hyperkalemia 3.9(3.5-4.3) mmol/L (*P*<0.001). Hyperkalemia occurred more frequently in older patients with a higher white blood cell (WBC) count, serum creatinine level, BUN, MELD score, and CLIF-SOFA score (*P*<0.001) and lower mean arterial pressure, serum sodium and albumin levels (*P*<0.05). These patients also had higher incidence of hepatic encephalopathy, bacterial infections, acute kidney injury, and gastrointestinal bleeding. Interestingly, no significant differences in the use of potassium-containing preparations, mainly spironolactone, potassium chloride, and potassium citrate, were observed among patients with and without hyperkalemia (*P*>0.05).

### 3.2. Correlation of Serum Potassium with Parameters of Renal Function

**Figure 2:**
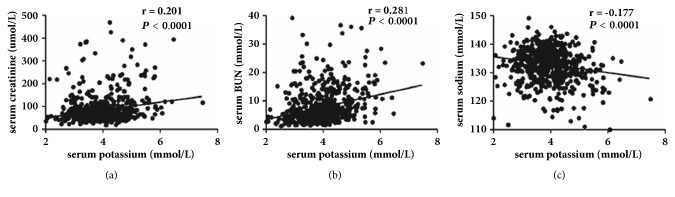
Correlations between serum potassium and serum creatinine, serum BUN, and serum sodium in patients with acute-on-chronic liver failure.

A positive correlation between serum potassium and both serum creatinine (r=0.201,* P*<0.001) and serum BUN (r=0.281,* P*<0.001) was detected in ACLF patients. By contrast, serum potassium had a negative correlation with serum sodium (r= -0.177,* P*<0.001) (Figure 2).

### 3.3. Independent Risk Factors Associated with the Presence of Hyperkalemia in ACLF Patients

Multivariate logistic regression analysis showed that independent factors of hyperkalemia were baseline serum potassium levels (odds ratio [OR]=3.48, 95%confidence interval[CI]=2.309-5.235,* P*<0.001) and the presence of acute kidney injury (OR=4.77, 95%CI= 2.479-9.166, and* P*<0.001) (Table 2).

### 3.4. Relationship between Hyperkalemia and Acute Kidney Injury in ACLF Patients

Hyperkalemia was more prevalent in AKI patients (23.6%) than in non-AKI patients (4.6%) (*P*<0.001). No significant differences in age, use of medications, etiology and complications of cirrhosis, WBC, ALB, INR, and parameters of liver function were observed among AKI patients with and without hyperkalemia(*P*>0.05). Of note, AKI patients with hyperkalemia had higher serum potassium values and lower TBIL values than AKI patients without hyperkalemia (*P*<0.05) (Table 3).

### 3.5. Effects of Hyperkalemia on 90-Day Survival in ACLF Patients

**Figure 3:**
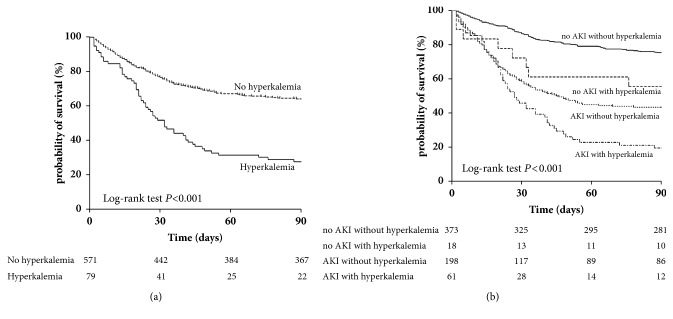
Kaplan-Meier survival curves (a) in patients with and without hyperkalemia (b) in patients with hyperkalemia according to presence of acute kidney injury (AKI).

Incidence of 90-day mortality in patients with and without hyperkalemia was 72.2% and 35.7%, respectively (*P*<0.001) (Figure 3(a)). In patients with hyperkalemia, nonsurvivors had a higher incidence of hepatic encephalopathy and acute kidney injury than survivors (*P*<0.001). Moreover, nonsurvivors had a higher INR values, serum potassium concentrations, and MELD scores than survivors (*P*<0.05) (Table 4).

In patients with hyperkalemia, a multivariate logistic regression analysis was used to evaluate independent factors associated with 90-day mortality. Based on the results of the univariate analysis of parameters presented in Table 4, the independent factors associated with 90-day mortality were high INR values (OR=8.19, 95%CI=1.668-40.202, and* P*=0.010) and acute kidney injury (OR=11.36, 95%CI=1.838-70.186, and* P*< 0.009) (Table 5(a)).

Due to the close association between hyperkalemia and acute kidney injury, we further stratified the 90-day mortality of patients with hyperkalemia according to the presence of acute kidney injury. The corresponding survival curves of AKI and non-AKI patients according to incident hyperkalemia are shown in Figure 3(b). The 90-day mortality rates of non-AKI patients with and without hyperkalemia were 44.4% and 24.7%, respectively (*P*<0.001). By contrast, the 90-day mortality rates of AKI patients with and without hyperkalemia were 80.3% and 56.6%, respectively (*P*<0.001).

A multivariate logistic regression analysis of all ACLF patients showed that HE, gastrointestinal bleeding, AKI and hyperkalemia, elevated TBIL and INR values, high MELD, and CLIF-SOFA scores were independent risk factors for predicting the 90-day mortality, with ORs of 3.34, 2.00, 2.35, 2.98, 1.00, 1.77, 0.90, and 1.19, respectively (Table 5(b)).

## 4. Discussion

Hyperkalemia is one of the most serious electrolyte disturbances with an associated sudden cardiac death and fatal arrhythmias. Under normal conditions, serum potassium concentration is strictly regulated by redundancy of homeostatic mechanism and maintained within a narrow range of 3.5-5.5mmol/L. Once these homeostatic mechanisms are disrupted, potassium abnormalities may occur. With the increased in serum potassium concentrations, the risk of adverse outcome increases substantially, which makes hyperkalemia a medical emergency that need special attention. However, in clinical practice, no single threshold could identify the imminent adverse outcome of hyperkalemia in different patients [19].

In our study, 12.2% ACLF patients developed hyperkalemia during hospitalization. Hyperkalemia commonly affects older patients with a higher WBC count, more severe hepatorenal function, and higher incidence of hepatic encephalopathy, bacterial infections, gastrointestinal bleeding and AKI. Patients with advanced cirrhosis manifest a hyperdynamic circulatory state, which is a progressive vasodilatory syndrome and a key factor in the pathogenesis of various complications of cirrhosis, prominently in the kidney [20]. Vasodilatation leads to a decreased effective arterial blood volume and activation of neurohumoral systems such as RAAS. Increased plasma antidiuretic hormone (ADH) levels lead to water and sodium retention causing ascites and dilution hyponatremia further [4]. In hyponatremia, the Na^+^-K^+^-ATPase pumps sodium ions out of the cell and potassium into the cell [21]. In addition, patients with cirrhosis are characterized by a state of secondary hyperaldosteronism. Aldosterone, a mineralocorticoid hormone, is known to regulate the sodium reabsorption and potassium secretion in renal cortical-collecting duct. Aldosterone acts by increasing the number of open sodium channels, leading to increased sodium reabsorption and potassium secretion. This mechanism explains the negative relationship between serum potassium and serum sodium in ACLF patients.

Gastrointestinal bleeding, increased diuretic doses, large volume paracentesis, and lactulose-induced diarrhea may lead to a state of hypovolemia in ACLF patients. Hypovolemic activates the renin-angiotensin system [21]. The increased circulating aldosterone stimulates renal sodium retention and affects potassium excretion. This perturbed potassium homoeostasis is further disturbed by pharmacologic agents such as antialdosterones and inhibitors of RAAS, which are known to affect serum potassium. Therefore, hyperkalemia can be considered a late event in the natural history of cirrhosis, which occurs after the onset of ascites, gastrointestinal bleeding, and renal impairment [4]. Of note, diuretics such as spironolactone, and various potassium-containing preparations (potassium chloride, potassium citrate, and potassium magnesium aspartate) are commonly used in the management of cirrhotic patients and may lead to hyperkalemia. However, in our study, no significant differences in the use of potassium-saving drugs were observed among patients with and without hyperkalemia (*P*>0.05).

We also found that higher baseline serum potassium levels and the presence of AKI were the independent risk factors of hyperkalemia. We further analyzed the relationship between hyperkalemia and AKI. We found that hyperkalemia was more frequently seen in patients with AKI. Surprisingly, no significant differences in age, etiology, and complications of cirrhosis and other parameters except for the higher baseline serum potassium concentration were observed among AKI patients with and without hyperkalemia on admission. Hence, it is difficult for clinicians to identify the early presence of hyperkalemia in AKI patients. However, the 90-day mortality was significantly higher in AKI patients with hyperkalemia than in non-AKI patients without hyperkalemia. Moreover, hyperkalemia is the independent risk factor for the 90-day mortality of ACLF patients. The physiopathologic association between the hyperkalemia and poor prognosis is complex and remains to be elucidated. The mechanisms that drive this poor prognosis are likely due to the occurrence of a severe disease and the use of therapeutic drugs, which in cases with a renal dysfunction may result in hyperkalemia [22]. This result is in line with our findings, indicating the positive relationship between serum potassium and creatinine as well as BUN, which are known markers of renal dysfunction. Hence, it reminds us that appropriate surveillance regimens and the timely correction of abnormal serum potassium levels and renal function are critical, especially in AKI patients.

This study had limitations. Firstly, this was a retrospective and single center study; the results need to be validated in multicenter, prospective study. Secondly, this study may underestimate the incidence and risk of potassium variations in clinical practice, where laboratory surveillance is frequently less assiduous and regular.

In conclusion, 12.2% of ACLF patients had hyperkalemia, and a close association between hyperkalemia and AKI was detected in ACLF patients. Hyperkalemia is more frequently seen in patients with AKI. The 90-day mortality rates were clearly different among AKI patients with and without hyperkalemia. AKI patients with hyperkalemia had the worst outcome. HE, gastrointestinal bleeding, AKI, hyperkalemia, elevated TBIL and INR values, and high MELD and CLIF-SOFA scores were independent factors for predicting the 90-day mortality in ACLF patients. The dynamic monitoring and timely treatment of abnormal serum potassium levels and renal function are critical for improving the patients' prognosis.

## Figures and Tables

**Table 1:** Characteristics of all patients according to the presence of hyperkalemia.

variables	without hyperkalemia	With hyperkalemia	*P*-value
	N=571	N=79	
Age (years)	51.0 ± 11.7	53.7 ± 11.0	0.028
Male-n%	436(76.4)	65(82.3)	0.241
Diabetes-n%	104(18.2)	15(19.0)	0.868
Hypertension-n%	74(13.0)	13(16.5)	0.392
**Etiology of cirrhosis-n (**%**)**			
Hepatitis B	327(57.3)	34(43.0)	0.017
Alcoholic	155(27.1)	35(44.3)	0.002
Hepatitis B plus alcoholic	30(5.3)	5(6.3)	0.896
Others	59(10.3)	5(6.3)	0.263
**Medications-n**%			
Diuretic (frusemide/spironolactone)	493(86.3)	68(86.1)	0.993
Potassium chloride	263(46.1)	33(41.8)	0.473
Potassium citrate granules	279(48.9)	34(43.0)	0.332
Beta-blockers	37(6.5)	5(6.3)	0.959
**Complications of cirrhosis-n**%			
Ascites	473(82.8)	67(84.8)	0.661
Bacterial infection	458(80.2)	74(93.7)	0.004
Hepatic encephalopathy	173(30.3)	33(41.8)	0.040
Acute kidney injury	198(34.7)	61(77.2)	<0.001
Gastrointestinal bleeding	111(19.4)	28(35.4)	0.001
**Clinical and laboratory data at admission**			
MAP(mmHg)	89.6 ± 12.2	84.7 ± 15.4	0.001
Heart rate(beats/minute)	84.2 ± 14.7	85.0 ± 16.6	0.658
WBC(×10^9^/L)	7.7 ± 4.9	9.9 ± 6.6	<0.001
PLT(×10^9^/L)	78.0(51.0-123.5)	98.5(48.0-130.0)	0.589
ALB(g/L)	28.3 ± 5.2	26.9 ± 5.8	0.026
TBIL(*μ*mol/L )	216.9(130.2-321.2)	144.2(104.5-299.7)	0.128
INR	2.1(1.7-2.6)	2.2(1.8-2.9)	0.177
BUN(mmol/L)	5.4(4.0-8.9)	9.6(5.2-14.4)	<0.001
Serum Cr(*μ*mol/L)	61.0(49.0-84.0)	87.0(58.0-122.0)	<0.001
Serum Na^+^ (mmol/L)	134.1(130.3-137.1)	131.3(125.1-135.6)	<0.001
Serum K^+^ (mmol/L)	3.9(3.4-4.3)	4.7(3.7-5.5)	<0.001
CTP score	12.0(10.0-13.0)	12.0(11.0-13.0)	0.066
MELD score	21.9 ± 7.1	25.1 ± 8.8	<0.001
CLIF-SOFA score	7.0(6.0-8.0)	7.0(7.0-11.0)	0.002
Number of deaths-n%	204(35.7)	57(72.2)	<0.001

MAP, mean arterial pressure; WBC, white blood cells; PLT, platelet; ALB, albumin; TBIL, total bilirubin; INR, international normalized ratio; BUN, blood uria nitrogen; Cr, creatinine; CTP, Child-Turcotte-Pugh; MELD, Model for End-Stage Liver Disease; CLIF-SOFA, chronic liver failure-sequential organ failure assessment.

**Table 2:** Risk factors for the presence of hyperkalemia in patients with acute-on-chronic liver failure.

Effect	Estimate	OR(95%CI)	Standard error	Wald X2	*P*-value
Acute kidney injury	1.562	4.77(2.479-9.166)	0.334	21.907	<0.001
Admission potassium levels (mmol/L)	1.246	3.48(2.309-5.235)	0.209	35.622	<0.001

**Table 3:** Characteristics of ACLF patients with acute kidney injury according to presence of hyperkalemia.

Variables	NO AKI		AKI		*P∗*-value
	No hyperkalemia	Hyperkalemia	No hyperkalemia	Hyperkalemia	
	N=373	N=18	N=198	N=61	
Age (years)	50.1 ± 11.7	50.1 ± 12.7	52.8 ± 11.5	54.8 ± 10.3	0.115
Male-n%	281(75.3)	15(83.3)	155(78.3)	50(82.0)	1.000
Diabetes-n%	63(16.9)	3(16.7)	39(19.7)	11(18.0)	1.000
Hypertension-n%	45(12.1)	4(22.2)	30(15.2)	9(14.8)	0.479
**Etiology of cirrhosis-n (**%**)**					
Hepatitis B	87(23.3)	7(18.8)	83(41.9)	28(45.9)	0.599
Alcoholic	231(61.9)	11(2.9)	80(40.4)	23(37.7)	0.078
Hepatitis B plus alcoholic	23(6.2)	0	9(4.5)	5(8.2)	0.583
Others	32(8.6)	0	26(13.1)	5(8.2)	0.583
**Medications-n**%					
Diuretic (frusemide /spironolactone)	309(82.8)	14(77.8)	183(92.4)	55(90.2)	0.324
Potassium chloride	155(41.6)	5(27.8)	108(54.5)	28(45.9)	0.171
Potassium citrate granules	184(49.3)	7(38.9)	95(48.0)	27(44.3)	0.686
Beta-blockers	22(5.9)	0	15(7.6)	5(8.2)	0.583
**Complications of cirrhosis-n**%					
Ascites	300(80.4)	13(72.2)	173(87.4)	54(88.5)	0.090
Bacterial infection	273(73.2)	15(83.3)	185(93.4)	59(96.7)	0.075
Hepatic encephalopathy	93(24.9)	4(22.2)	79(39.9)	29(47.5)	0.063
Gastrointestinal bleeding	53(14.2)	3(16.7)	58(29.3)	25(41.0)	0.091
**Clinical and laboratory data at admission**					
MAP(mmHg)	82.3 ± 14.6	82.9 ± 11.2^■^	86.8 ± 13.9	85.2 ± 16.5	0.578
Heart rate(beats/minute)	91.2 ± 11.0	81.1 ± 19.4	87.7 ± 14.4	86.2 ± 15.7	0.269
WBC(×10^9^/L)	6.8 ± 4.0	6.5 ± 4.1	9.4 ± 5.8	10.9 ± 6.8	0.010
PLT(×10^9^/L)	81.0(51.0-126.0)	75.5(31.8-151.3)	72.5(49.8-115.0)	90.0(55.0-126.0)	0.444
ALB(g/L)	29.1 ± 5.2	28.5 ± 5.5	26.8 ± 5.0	26.4 ± 5.8	0.177
TBIL(*μ*mol/L )	210.4(131.3-290.7)	281.5(106.7-343.6)	225.5(117.2-360.9)	168.6(104.2-268.3)^▲^	0.086
INR	2.0(1.7-2.5)	2.3(1.8-3.4)	2.2(1.7-2.9)	2.2(1.8-2.8)	0.532
BUN(mmol/L)	4.8(3.6-6.5)	5.2(3.6-8.4)	8.9(5.1-14.2)	10.5(6.0-16.2)	0.002
Serum Cr(*μ*mol/L)	55.0(46.0-67.0)	63.5(53.8-90.3)^■^	88.0(63.0-139.0)	95.0(67.5-128.0)	0.013
Serum Na^+^ (mmol/L)	134.7(131.7-137.5)	132.8(135.0-137.0)	132.3(127.9-135.6)	130.4(125.0-135.3)	0.516
Serum K^+^(mmol/L)	3.9(3.4-4.2)	4.2(3.6-5.6)^■^	3.9(3.4-4.3)	4.8(3.8-5.5)^▲^	0.599
CTP score	11.0(10.0-12.0)	12.0(10.0-13.0)	12.0(11.0-13.0)	12.0(11.0-13.0)	0.398
MELD score	19.8 ± 5.7	24.5 ± 7.6^■^	25.9 ± 8.0	25.2 ± 9.1	0.767
CLIF-SOFA score	7.0(6.0-8.0)	7.5(7.0-11.3)^■^	8.0(7.0-10.0)	7.0(6.5-11.0)	0.275

***P*** **∗** **value between patients with and without AKI with hyperkalemia; *P*** ^■^ **<0.05 patients without AKI no hyperkalemia versus hyperkalemia; *P*** ^▲^ **<0.05 patients with AKI no hyperkalemia versus hyperkalemia.**

MAP, mean arterial pressure; WBC, white blood cells; PLT, platelet; ALB, albumin; TBIL, total bilirubin; INR, international normalized ratio; BUN, blood urea nitrogen; Cr, creatinine; CTP, Child-Tturcotte-Pugh; MELD, Model for End-Stage Liver Disease; CLIF-SOFA, chronic liver failure-sequential organ failure assessment.

**Table 4:** Comparison between patients with hyperkalemia who died or survived.

**Variables**	**Survival**	**Non-survival**	***P*-value**
	**N=22**	**N=57**	
Age (years)	52.5 ± 7.9	54.2 ± 12.0	0.563
Male-n%	17(77.3)	48(84.2)	0.469
**Etiology of cirrhosis-n (**%**)**			
Hepatitis B	7(31.8)	23(40.4)	0.484
Alcoholic	12(54.5)	26(45.6)	0.476
Hepatitis B plus alcoholic	1(4.5)	4(7.0)	1.000
others	2(9.1)	3(5.3)	0.614
**Medications-n**%			
Diuretic (frusemide/spironolactone)	20(90.9)	48(84.2)	0.683
Potassium chloride	11(50.0)	22(38.6)	0.357
Potassium citrate granules	9(40.9)	25(43.9)	0.812
Beta-blockers	1(4.5)	4(7.0)	1.000
**Complications of cirrhosis-n**%			
Ascites	17(77.3)	50(87.7)	0.246
Bacterial infection	20(90.9)	54(94.7)	0.614
Hepatic encephalopathy	5(22.7)	28(49.1)	0.033
Acute kidney injury	12(54.5)	49(86.0)	0.003
Gastrointestinal bleeding	5(22.7)	23(40.4)	0.142
**Clinical and laboratory data at admission**			
MAP(mmHg)	62.6 ± 15.7	83.7 ± 15.9	0.327
Heart rate(beats/minute)	81.8 ± 11.7	86.3 ± 18.0	0.292
WBC(×10^9^/L)	8.5 ± 4.8	10.5 ± 7.1	0.248
PLT(×10^9^/L)	104.0(72.5-152)	77.0(47.0-120.5)	0.056
ALB(g/L)	28.4 ± 3.7	26.3 ± 6.3	0.151
TBIL(*μ*mol/L )	228.8(104.4-343.6)	184.3(106.6-286.9)	0.314
INR	1.8(1.6-2.1)	2.5(1.8-3.3)	0.003
BUN(mmol/L)	6.6(4.3-10.8)	10.5(5.6-15.3)	0.057
Serum Cr(*μ*mol/L)	70.6(54.0-104.0)	95.0(60.0-129.0)	0.077
Serum Na^+^ (mmol/L)	132.2(124.6-137.1)	131.0(125.2-135.6)	0.600
Serum K^+^ (mmol/L)	3.8(3.4-5.5)	4.9(4.0-5.6)	0.038
CTP score	11.5(10.0-13.0)	12.0(11.0-13.0)	0.079
MELD score	22.1 ± 4.4	26.2 ± 9.7	0.016
CLIF-SOFA score	7.0(7.0-10.0)	8.0(7.0-11.5)	0.596

MAP, mean arterial pressure; WBC, white blood cells; PLT, platelet; ALB, albumin; TBIL, total bilirubin; INR, international normalized ratio; BUN, blood urea nitrogen; Cr, creatinine; CTP, Child-Turcotte-Pugh; MELD, Model for End-Stage Liver Disease; CLIF-SOFA, chronic liver failure-sequential organ failure assessment.

**(a):** 

**Effect**	**Estimate**	**OR (95**%**CI)**	**Standard error**	**Wald X2**	***P*-value**
Acute kidney injury	2.43	11.36(1.838-70.186)	0.929	6.837	0.009
INR	2.103	8.19(1.668-40.202)	0.812	6.707	0.010

**(b):** 

**Effect**	**Estimate**	**OR (95**%**CI)**	**Standard error**	**Wald X2**	***P*-value**
Hepatic encephalopathy	1.206	3.34(2.146-5.195)	0.226	28.560	<0.001
Gastrointestinal bleeding	0.693	2.00(1.207-3.315)	0.258	7.231	0.007
Acute kidney injury	0.853	2.35(1.482-3.714)	0.234	13.241	<0.001
Hyperkalemia	1.091	2.98(1.539-5.760)	0.337	10.505	0.001
TBIL	0.004	1.00(1.002-1,006)	0.001	13.893	<0.001
INR	0.568	1.77(1.230-2.532)	0.184	9.526	0.002
MELD	-0.110	0.90(0.826-0.973)	0.042	6.888	0.009
CLIF-SOFA	0.176	1.19(1.065-1.335)	0.058	9.307	0.002

INR, international normalized ratio; TBIL, total bilirubin; MELD, Model for End-Stage Liver Disease; CLIF-SOFA, chronic liver failure-sequential organ failure assessment.

## Data Availability

All data are available on the website of Chinese clinical trial registry within six months after the manuscript published.

## Abbreviations

ACLF: Acute-on-chronic liver failure
AKI: Acute kidney injury
CTP: Child-turcotte-pugh
MELD: Model for End-Stage Liver Disease
CLIF-SOFA: Chronic liver failure-sequential organ failure assessment
HE: Hepatic encephalopathy
WBC: White blood cells
PLT: Platelet
ALB: Albumin
TBIL: Total bilirubin
BUN: Blood urea nitrogen
Cr: Creatinine
INR: International normalized ratio.
